# Injectability of Thermosensitive, Low-Concentrated Chitosan Colloids as Flow Phenomenon through the Capillary under High Shear Rate Conditions

**DOI:** 10.3390/polym12102260

**Published:** 2020-10-01

**Authors:** Anna Rył, Piotr Owczarz

**Affiliations:** Department of Chemical Engineering, Lodz University of Technology, 90-924 Lodz, Poland; piotr.owczarz@p.lodz.pl

**Keywords:** injectable hydrogels, chitosan scaffold, hypodermic needles, capillary rheometry, in situ gelling liquids, Cox–Merz rule, shear-induced polymer deformation

## Abstract

Low-concentrated colloidal chitosan systems undergoing a thermally induced sol–gel phase transition are willingly studied due to their potential use as minimally invasive injectable scaffolds. Nevertheless, instrumental injectability tests to determine their clinical utility are rarely performed. The aim of this work was to analyze the flow phenomenon of thermosensitive chitosan systems with the addition of disodium β-glycerophosphate through hypodermic needles. Injectability tests were performed using a texture analyzer and hypodermic needles in the sizes 14G–25G. The rheological properties were determined by the flow curve, three-interval thixotropy test (3ITT), and Cox–Merz rule. It was found that reducing the needle diameter and increasing its length and the crosshead speed increased the injection forces. It was claimed that under the considered flow conditions, there was no need to take into account the viscoelastic properties of the medium, and the model used to predict the injection force, based solely on the shear-thinning nature of the experimental material, showed very good agreement with the experimental data in the shear rate range of 200–55,000 s^−1^. It was observed that the increase in the shear rate value led to macroscopic structural changes of the chitosan sol caused by the disentangling and ordering of the polysaccharide chains along the shear field.

## 1. Introduction

In recent years, due to the growing number of traffic accidents and surgical interventions, innovative methods of treatment and regeneration of damaged tissues are sought. An interesting solution seems to be injectable hydrogels [1,2,3] that are obtained from synthetic and natural polymers. These materials, characterized by the three-dimensional architecture, have the ability to mimic the extracellular matrix [4,5,6], and thus support cell proliferation. One of the desired feature of the injectable hydrogels is their shear-thinning behavior [7,8], resulting in a reduction in viscosity as they flow through the needle. Thanks to this, they can be precisely designed in a laboratory and then successfully injected into the body.

Although the injection application reduces invasiveness compared to implant scaffolds, one of its biggest challenges is reducing the patient’s discomfort during drug administration [9]. In addition to purely pharmaceutical aspects such as the use of anesthetics, the reduction of injection pain is possible by reducing the diameter of the needle. However, this causes a significant change in the flow conditions of the medium, an increase in the flow resistance in the needle, and consequently, an increase in the force needed for injection. In extreme cases, choosing a needle that is too small may make an injection impossible or lead to an uncontrolled, rapid release of substances inside the body. Therefore, it is necessary to optimize the injection process in terms of the drug physicochemical properties, the diameter of the needles used, as well as the injection speed.

When designing minimally invasive hydrogel polymer matrices, apart from their shear-thinning nature, it is also worth paying attention to their gelation ability under the influence of changes in environmental conditions, e.g., temperature [10,11]. In the case of such systems, after exceeding the critical solution temperature (CST), they undergo a thermodynamically non-equilibrium sol–gel phase transition, forming an infinite three-dimensional structure. This process can be observed during both cooling and heating, and the materials are characterized by an upper critical solution temperature (UCST) and a lower critical solution temperature (LCST) [11], respectively. Although biopolymer-based hydrogels formed under the influence of cooling are much more common, the systems characterized by LCST seem to be more interesting due to their possible application in the form of a sol at room temperature and the formation of a matrix directly at the site of the disease inside the body. An example of such systems are chitosan hydrogels with the addition of glycerophosphate salt, which simultaneously exhibit shear-thinning rheological behavior. Thermosensitive chitosan hydrogels are often investigated both in terms of the gelation mechanism [12,13,14,15,16,17,18,19] and their application potential [20,21,22,23]. The latter mostly focuses on the study of biocompatibility, cytotoxicity, and bioactivity, and are based on cell cultures [21,24,25] or laboratory tests on small animals [26,27].

**Figure 1:**
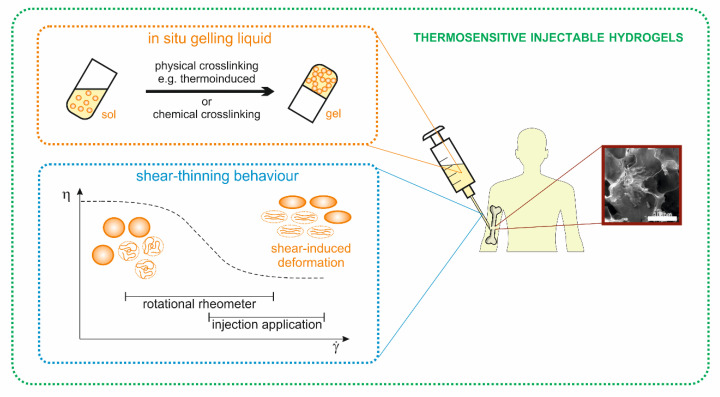
Graphical interpretation of the synergistic properties of thermosensitive injectable hydrogels.

In addition to the desired characteristics for all materials used in tissue engineering such as biodegradability, biocompatibility, and non-toxicity, thermosensitive injection hydrogels (Figure 1) must also be injectable. For this purpose, the designed system at room temperature should remain in the liquid-like sol phase. Such systems, called in situ gelling liquids, form a gel after administration via physical or chemical cross-linking [8,28].

Despite many studies on injectable materials, instrumental studies, which consist of recording the load necessary to maintain the movement of a syringe plunger as a function of the distance traveled, are surprisingly rare. The characteristic courses of the determined injection curves depend on the properties of the tested medium, especially its viscosity [9,29,30]. Based on the obtained experimental data, the following values are determined: the initial glide force (IGF) necessary to initiate the plunger movement, the dynamic glide force (DGF) necessary to maintain the plunger movement during injection, and the maximum force value recorded during the measurement [29,30,31]. Zhang et al. [31], in their research, proposed to use the parameter called total work done as a quantity better than the above-mentioned forces describing the injectability process. Moreover, the use of instrumental injectability tests enables repeatable quantitative analysis, the results of which could be successfully correlated with panel tests [29]. Consequently, their conduct allows for the characterization of the medium by the laboratory tests, changes in its composition in order to improve injectability, or a precise recommendation of administration conditions (suggested needles, temperature, etc.).

So far, the results of injectability studies conducted for Carbopol [32], hyaluronic acid [33], shear-thinning hydrogels, (Hydroxypropyl)methyl cellulose (HPMC) and polyethylene oxide (PEO) [31] polymer solutions, immunoglobin solutions, monoclonal antibodies [34], protein systems [32], and other specific pharmaceuticals [9,35] have been published. In most of them, researchers focus mainly on determining the effect of substance concentration on its injectability process. Sometimes they correlated the obtained experimental data with the viscosity of the medium or described the injectability process using needles differing in sizes. Extremely interesting results were presented by Rungseevijitprapa and Bodmeier [30]. They comprehensively discussed the injection process of microparticle systems including the injection site effect. The authors unequivocally showed that the forces required to inject “into the air” were much lower than those required for subcutaneous and intramuscular injections.

The injectability of the hydrogels undergoing a thermally induced sol–gel phase transition is almost exclusively determined by the LCST value. This results from the assumption that the samples remain in the sol phase during injection, determined by the advantage of viscous properties over elastic ones. For this purpose, the LCST value should be slightly higher than room temperature, but not exceed human body temperature. Despite the lack of instrumental tests, many authors clearly indicate the possible injection application [3,18,36,37,38] of the investigated systems solely based on their rheological properties. Although these features will strongly determine the possibility of the injectability process, determination of the forces required for injection is essential for assessing their clinical utility [33]. A small number of published papers on thermosensitive chitosan hydrogels mainly presented the influence of the molecular weight of the polymer [39] and the addition of calcium phosphate ceramics [40,41] on their injectability. In these studies, one size of the injection needle was used, or even tests without a needle were performed.

The majority of the studies on the injection phenomenon use the theoretical basis of capillary rheometry of non-Newtonian fluids to define the flow conditions in the needle (shear rate values). Such tests are carried out to determine the viscosity of the investigated medium beyond the measuring range of the rotational rheometer [34,35,42]. These analyses are very rarely used to predict the forces required for application and to correlate them with experimental data. Allmendinger et al. [32] proposed a formula to predict the forces required for injection, taking into account the non-Newtonian nature of the experimental medium that was verified with the results of instrumental tests. In their considerations, the authors introduced a new quantity called effective shear rate, the value of which was lower than the corrected shear rate value. Despite a comprehensive discussion, the authors did not consider the possible wall slip effect, which would explain the lower values of the shear rate.

During the flow of long-chain polymer solutions through the capillaries, their conformation changes [43,44]. The phenomenon of their deformation and ordering along the streamlines determines their shear-thinning rheological behavior, the intensity of which depends on the degree of entanglement of the polymer coils. Xu et al. [45] showed that, with increasing shear rate, the end-to-end distance of the polymer chain increases, indicating its disentanglement. This phenomenon was also observed during rheometric measurements with simultaneous analysis using the small-angle light scattering technique [46,47,48].

In the case of melted polymers and their solutions, the Cox–Merz rule is used to assess the relationship between viscous and viscoelastic properties related to the existence of an internal polymer structure. It is assumed that this rule is met if the apparent viscosity obtained from steady shear and complex viscosity from oscillatory measurements are equal. Although the exact physical mechanism determining the validity of this rule is unknown and is based solely on experimental data, according to the considerations by Bair et al. [49], the strong relationship between shear stress and chain conformation plays a crucial role.

The aim of this work was to analyze the flow phenomenon of thermosensitive, low-concentrated chitosan colloids with the addition of disodium β-glycerophosphate through injection needles under high shear rate conditions. The analysis will be carried out using the classical relationships of capillary rheometry of non-Newtonian fluids. The phenomena of laminar flow under high shear rate conditions, leading to flow resistance and deformation of long-chain polymer particles, will be discussed. The obtained results of the experimental tests will be compared with the theoretically determined values of the forces necessary for injection, taking into account the non-Newtonian nature of the medium as well as the influence of their viscoelastic properties.

## 2. Materials and Methods

### 2.1. Preparation of Injectable Chitosan Hydrogels

Thermosensitive chitosan hydrogels were prepared in accordance with the commonly used methodology proposed by Chenite et al. [18]. For this purpose, 0.4 g of crab-derived chitosan (deacetylation degree: 81.8%, molecular weight: 680 kDa, CAS No. 9012-76-4) was dissolved in 16 mL of 0.1 M hydrochloric acid (CAS No. 7647-01-0) and left for 24 h in room temperature. Next, a suspension of disodium β-glycerophosphate (CAS No. 13408-09-8) was prepared by dispersing 2 g of the powder in 2 mL of distilled water. After cooling both samples for 2 h at 5 °C, the glycerophosphate suspension was introduced drop by drop into chitosan.

All materials were provided by the Sigma-Aldrich^®^ company (Poznan, Poland) and used without further treatment.

### 2.2. Instrumental Injectability Studies

Instrumental injectability tests were carried out using a Brookfield CT3 texture analyzer (London, United Kingdom) with a 4.5 N load cell (44 N) in compression mode. In the study, hypodermic needles (Zarys International Group, dispoFINE, Zabrze, Poland) in sizes 14G–25G (Table 1) and 2 mL disposable syringes (Braun Injekt, ALMO-Erzeugnisse Erwin Busch GmbH, Bad Arolsen, Germany) with an internal diameter of 9.75 mm filled with 0.5 mL of chitosan sol were used.

During the measurements, the force as a function of the distance traveled by the analyzer head was recorded. The injectability process was carried out “into the air”. The detailed measurement procedure is described below. Three repetitions of the measurement were made; sample was injected immediately after being taken out of the refrigerator to ensure a constant temperature of the sol.

The injectability was discussed based on the determined values of dynamic glide force (DGF), the application of which is necessary to maintain the syringe plunger movement. Its value was determined on the basis of the average value of the longest range of forces in which they assume a constant value [31].

#### 2.2.1. Influence of the Injection Needle Used (Diameter and Length)

In order to determine the influence of the used needle on the injection process, needles with sizes 14G–25G were used. The effect of needle length was determined on the basis of tests carried out with the use of 23G needles with a length of 25 mm or 30 mm. The crosshead speed of 1 mm·s^−1^ was used, which is the most commonly used rate for manual injection [31,40].

#### 2.2.2. Influence of the Injection Rate

To discuss the injection rate effect, a 22G hypodermic needle was used. Its choice resulted from the appropriate length-to-diameter ratio, which eliminates the influence of the inlet and end effects [35] and ensures fully developed fluid flow. During the measurements, the crosshead speeds in the range of 0.2–1 mm·s^−1^ were used, corresponding to the possible speeds of manual injection.

#### 2.2.3. Injection under Constant Shear Conditions in the Needle

In order to test the injection process under constant shear rate conditions, the crosshead speed was varied according to the values given in Table 2. The selection of needles and the value of the crosshead speed resulted from the ability to precisely maintain the probe displacement parameters by the apparatus.

#### 2.2.4. Effect of Uncontrolled Storage at Room Temperature

For the same reasons as in Section 2.2.2, studies on the effect of the storage time of chitosan sol at room temperature were carried out with a 22G needle and a crosshead speed of 1 mm·s^−1^. The experimental material was injected immediately after being removed from the refrigerator and after storage for 5, 10, 15, 30, and 60 min at room temperature.

### 2.3. Rheological Characterization

The rheological characterization of the experimental medium was performed using an Anton Paar Physica series MCR 301 rotational rheometer (Anton Paar, Warszawa, Poland) equipped with a plate–plate measurement system (diameter 25 mm, gap 0.2 mm). A sandblasted plate (serial No. 63000) was used in the tests. Detailed measurement parameters were set depending on the test performed and are described below.

#### 2.3.1. Flow Curve in the Range of High Shear Rates

In order to determine the rheological properties of the chitosan sol, the flow curves in the shear rate range of 100–18,000 s^−1^ were determined. The lower range resulted from the possible values of the shear rate occurring in the 14G needle using the 1 mm·s^−1^ crosshead speed, and the upper range resulted from the limit of the test apparatus. The measurement was carried out at 5 °C.

#### 2.3.2. The Cox–Merz Rule

In order to determine the possible influence of the viscoelastic properties of the chitosan sol during the flow through the needles on additional flow resistances, studies were conducted to determine the relationship between purely viscous properties determined in rotational measurements with viscoelastic properties obtained from oscillatory measurements. During a series of measurements, flow curves were determined in the shear rate range of 10^−2^–10^3^ s^−1^ and frequency sweep tests in the range of 10^−2^–10^3^ rad·s^−1^ were performed. The measurements were carried out at temperatures of 5, 15, 25 and 37 °C, and each time the sample was changed after the test.

#### 2.3.3. Three-Interval Thixotropic Tests

In order to determine the effect of the short-term shear interval on the macroscopic viscoelastic properties of the chitosan sol, three-interval thixotropic tests were carried out in the oscillation-rotation-oscillation mode [15,50]. In the oscillatory shear intervals, constant deformation values of γ = 1% and ω = 5 rad·s^−1^ lasting 100 s and 600 s, respectively, were applied. The rotational shear interval lasted 3 s and the shear rate values varied in the range of 200–7000 s^−1^. Based on the experimental data, in particular, the value of the storage modulus *G’*, the degree of deformation and recovery of the polymer structure were determined depending on the shear rate value. The value of the deformation parameter (*Def*) was calculated according to the following equation:

(1) $$Def=\frac{G_{i}-G_{0}}{G_{i}}\times 100\%$$

where *G_i_* is the value of the storage modulus in the 1st oscillatory shear interval and *G*_0_ is the value of the storage modulus immediately after the 2nd rotational shear interval.

The value of the recovery parameter (*Rec*) was determined according to the formula:

(2) $$Rec=\frac{G_{30}}{G_{i}}\times 100\%$$

where *G*_30_ is the storage modulus *G’* value 30 s after the end of the 2nd shear interval.

## 3. Results

### 3.1. Rheological Characterization of Chitosan Sol

**Figure 2:**
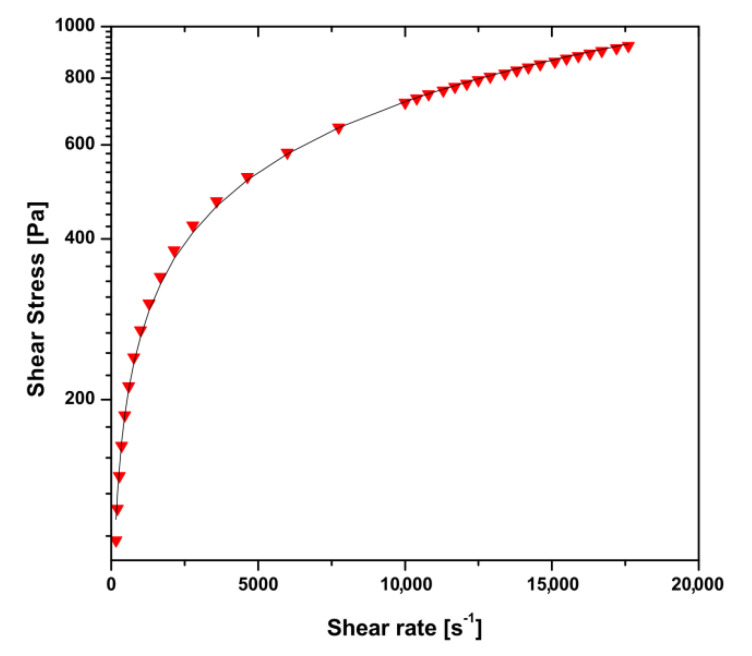
Flow curve of chitosan sol obtained at 5 °C.

Figure 2 shows the flow curve of chitosan sol obtained in the shear rate ranges observed in the injection needles used. The raw data were approximated by the Ostwald–de Waele power law. The values of the characteristic flow index (*n* = 0.44) and the consistency coefficient (*K* = 12.54 Pa·s^0.44^) were used in the model used to predict the force necessary for injection—Equation (6). The obtained parameters values of the power law proved a very strong shear-thinning behavior of chitosan sol under the flow conditions observed in injection needles.

### 3.2. Theoretical Analysis of Flow Conditions in an Injection Needle

In order to analyze the injection phenomenon, a proper discussion of the experimental data obtained during instrumental injectability tests must be preceded by a theoretical discussion of the flow conditions in the injection needle, which will be understood as a capillary. In the case of the considered flow of a non-Newtonian medium, its viscosity significantly depends on the value of the shear rate. Simultaneously, this value and the shear rate profile in the capillary significantly depend on the non-Newtonian nature of the experimental medium. In order to determine the real value of the shear rate considering the shear-thinned behavior of the chitosan sol, the correlation proposed by Rabinowitsch–Mooney was used:

(3) $$\dot{\gamma }=\frac{32\times Q}{\pi \times d^{3}\;}\times \left(\frac{3n+1}{4n}\right)$$

The above relationship, expressed as the crosshead speed *v_T_*, and taking into account the continuity equation, takes a more useful form:

(4) $$\dot{\gamma }=8\times v_{T}\times {\left(\frac{d_{syringe}}{d_{needle}}\right)}^{3}\times \left(\frac{3n+1}{4n}\right)$$

The determined values of the shear rate for the most commonly used crosshead speed (*v_T_* = 1 mm·s^−1^) and the value of the characteristic flow index (*n* = 0.44) are summarized in Table 1. It is worth noting that the obtained values were 32% higher compared with the Newtonian fluid (*n* = 1). This meant that the viscosity of the medium during flow will drop even more. Due to the small diameters and low velocities that are observed in capillaries, the nature of the fluid flow in them is usually described as laminar. In order to unequivocally determine the nature of the flow of chitosan sol in the range of the needles used, the values of the generalized Reynolds number proposed by Metzner and Reed were determined, in accordance with the following formula:

(5) $$Re_{MR}\;=\frac{\rho \cdot u_{needle}{}^{2-n}\times d_{needle}{}^{n}}{8^{n-1}\times K^{\prime }}$$

In the above relation, the consistency coefficient *K’* was determined on the basis of the parameters of the Ostwald–de Waele model. It was found that the calculated values of the generalized Reynolds number (Table 1) were much lower than the value of the critical Reynolds number *Re_cr_* = 2395 (for *n* = 0.44) determined from the Ryan and Johnson relationship. This meant that the flow of chitosan sol in injection needles was laminar, in some cases even creeping, thus no velocity vector components other than parallel to the flow direction were observed during the flow.

### 3.3. Instrumental Injectability Tests

**Figure 3:**
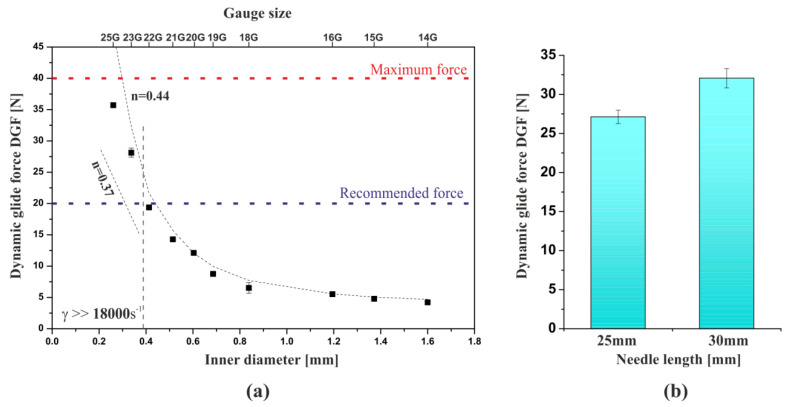
(**a**) Experimental and theoretical value of the force required for the injection depending on the inner diameter of the needle; (**b**) influence of the needle length on the value of the dynamic glide force (DGF).

Figure 3 shows the results of the injectability tests determining the effect of the needle diameter using a constant crosshead speed of 1 mm·s^−1^. It was found that the diameter of the used needle had a significant influence on the value of the force necessary to inject chitosan sol. With the decreasing inner diameter of the injection needle, the value of the determined DGF force increased significantly. From a practical point of view, it is recommended to use needles not smaller than 21G for the injection of colloidal chitosan sol. This is due to the determined values of the force necessary to maintain the plunger movement not exceeding 20 N [9,33]. The use of smaller needles, 22G and 23G, may exceed the recommended value of the dynamic force DGF, and the selection of needles smaller than 25G may exceed the maximum force of 40 N [9]. Attempting to use them may fail or lead to uncontrolled outflow of liquid and tissue damage. It should also be noted that the determined values of DGF forces during injection “into the air” will be lower than the actual values of forces during subcutaneous or intramuscular injection due to the occurrence of additional resistance of the tissue [30].

The difficulty in administration along with the decreasing diameter of the injection needle result from the increasing flow resistance, and thus the greater force necessary to overcome them. Since the flow through the hypodermic needle can be described using the theoretical basis of capillary rheometry of non-Newtonian fluids, an attempt was made to determine the value of the force necessary for injection in accordance with the following equation:

(6) $$N_{model}=F_{s}+\frac{8^{n}\times K^{\prime }\times L_{needle}\times u_{needle}{}^{n}\;}{d_{needle}{}^{n+1}}\times \pi \times d_{syringe}{}^{2}\;$$

The proposed equation was based on the determination of linear pressure drops during laminar flow of a non-Newtonian medium through a circular pipe. As shown in Figure 3a, the predicted force values according to Equation (6) described the experimental data satisfactorily. Reducing the needle diameter with the use of a constant crosshead speed will also increase the flow velocity in the capillary, thus causing even greater flow resistance.

The observed deviation of the experimental data from the theoretical force values (Equation (6)) for the smallest needles (23G and 25G) may result from an even stronger phenomenon of shear-thinning under the influence of very high shear rates. In these needles, the calculated values of the shear rate (Table 1) were higher than the measuring range of the rotational rheometer (18,000 s^−1^) with which the rheological parameters of the chitosan hydrogel were determined. In the considered range of shear rates, a second region of shear thinning may occur, where the flow behavior index *n* for long-chain polymers may even reach a value of 0.37 [45]. Consequently, a possible decrease in the value of the flow index parameter will result in a reduction of the injection force due to the lower flow resistance. On this basis, for needles smaller than 0.4 mm, where the shear rate exceeded 18,000 s^−1^, the area of the possible value of the force necessary for injection was determined within the limit value of the parameter *n* from 0.37 (determined in accordance with the literature data) to 0.44 (resulting from the rheometric measurements).

When any fluid flows through the syringe–needle system, there will be additional local resistances related to the narrowing of the diameter during the inflow of fluid into the needle as well as its outflow from the needle. However, due to their insignificant effect in total resistances (value determined based on the theoretical equations below 0.3%), these components may be neglected.

**Figure 4:**
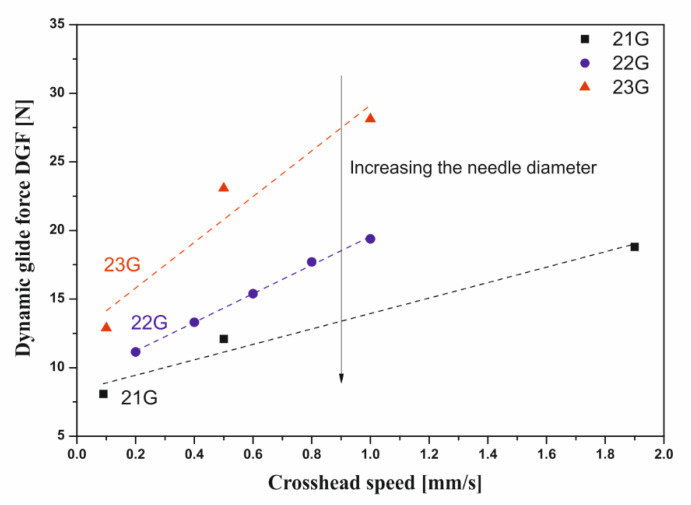
Influence of the crosshead speed on the value of the dynamic glide force (DGF) for needles 21G, 22G, and 23G.

Apart from the diameter of the injection needle, its length will also significantly affect the value of linear flow resistance. According to Equation (6), this value will increase in direct proportion to the capillary length. Figure 3b shows the results of instrumental tests confirming these dependencies. From a practical point of view, the analysis of the obtained data and theoretical relationships allowed us to indicate another reason, apart from purely clinical reasons, for using the shortest possible needles. Based on the data obtained, it can be seen that the DGF force increased proportionally with the increase in the crosshead speed (Figure 4).

Even though in Equation (6), there is no term related to the crosshead speed, this quantity will unequivocally define the volumetric flow rate and consequently, the flow velocity of the medium in the injection needle. This follows from the continuity equation for an incompressible fluid:

(7) $$Q\;=\frac{\pi \times d_{syringe}{}^{2}}{4}\times v_{T}=\frac{\pi \times d_{needle}{}^{2}}{4}\times u_{needle}$$

This means that, when considering the same syringe–needle system, a twofold increase in the crosshead speed will result in a twofold increase in the flow velocity through the injection needle. According to Equation (6), for shear-thinning fluids, the final influence of the flow velocity through the needle will be smaller because of the exponent *n* < 1 at the velocity component. Analyzing only the velocity value in the needle, it could be assumed that the dependence of the dynamic glide force on the velocity should be a power function with an exponent less than one. However, an increase in fluid flow velocity will cause an increase in the shear rate value and thus a decrease in the apparent viscosity value.

The influence of the crosshead speed on the injection force value discussed in the example of the 22G needle will be qualitatively met for all needles. According to the data presented in Figure 4, the smaller the injection needle, the stronger the impact of the crosshead speed on the DGF force values [35]. This dependence results directly from the proportionality constant in the continuity equation. In practice, this means that in the event of unforeseen difficulties during injection, a slower injection attempt may be sufficient, especially in the case of very small needles where the risk of exceeding the maximum allowable forces is much greater (Figure 3a).

**Figure 5:**
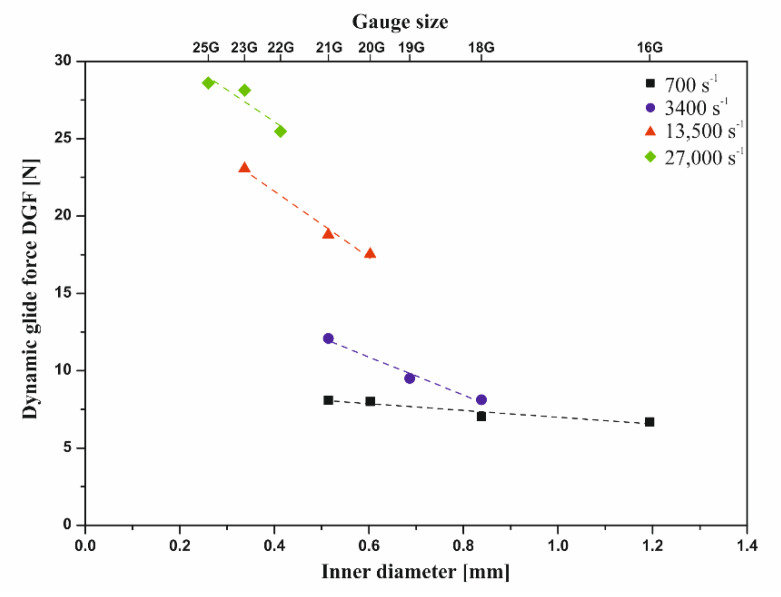
Influence of the needle diameter on the injection process under constant flow conditions expressed by the shear rate value.

In order to eliminate the different shear conditions during the flow of chitosan sol, measurements were carried out at a constant value of the shear rate. Thus, a constant value of the apparent viscosity of the chitosan sol was ensured during the flow through the considered injection needles. The determined values of the dynamic force DGF as a function of the internal diameter of the capillary used for the assumed constant values of the shear rate are shown in Figure 5.

The analysis of the obtained data showed that, regardless of the injection needle used, ensuring the same flow conditions in the range of low (in relation to injection) shear rates hardly affected the obtained DGF values. The higher the set values for the shear rate, the more the injection needle used affected the value of the DGF value. This was particularly observed in the case of injection needles 18G–21G for assumed shear rates of 700 s^−1^ and 3400 s^−1^. Thus, the conducted analysis confirmed the phenomena observed during the study of the influence of the crosshead speed (Figure 4), while limiting the impact of the variable value of apparent viscosity. 

**Figure 6:**
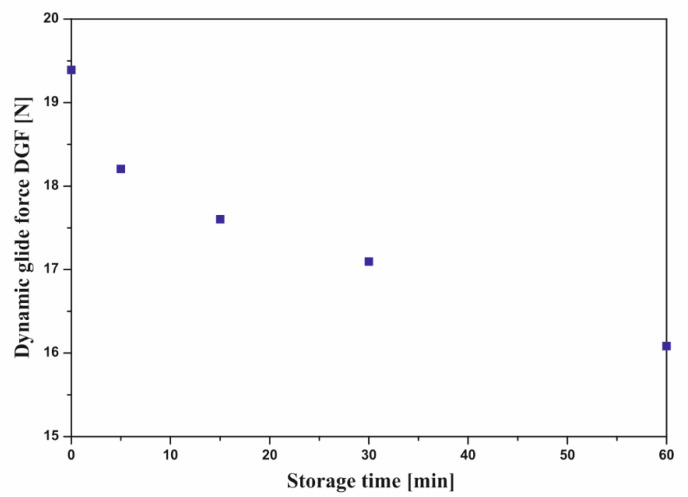
Effect of storage time at room temperature on injectability of chitosan sol.

In the case of thermosensitive hydrogels characterized by a lower critical solution temperature, uncontrolled storage of the sol at room temperature may be another important factor influencing the injection process. Figure 6 shows the determined values of dynamic glide force (DGF) as a function of the storage time of the sample at room temperature.

The analysis of the obtained experimental data indicated that storage of the samples before injection caused a decrease in the DGF value. It was found that storing chitosan sol at room temperature for 60 min before injection caused a reduction in the value by approximately 17% from 19.4 N to 16.1 N. This resulted from the decreasing apparent viscosity value caused by unforced heating of the sample. It is worth mentioning that the injection temperature was lower than the LCST value determined on the basis of previous rheological measurements [15,51], and the sol remained in the viscoelastic flow region. In this area, a decrease in viscosity with increasing temperature was observed. Therefore, there was no risk that uncontrolled storage of the thermosensitive chitosan hydrogel at room temperature for too long (up to 1 h) will result in the formation of a three-dimensional lattice and hinder the injection process.

However, it should be noted that in the case of using other systems with an LCST value close to room temperature [36], leaving the thermosensitive hydrogel for too long may lead to the formation of a polymer network, and thus a significant increase in the viscosity value, which will consequently make injection application difficult.

### 3.4. Influence of the Injection Application on the Conformation of Chitosan Molecules

According to Equation (6), the theoretical analysis of the forces required for injection was carried out taking into account the non-Newtonian nature of the chitosan sol described by the power model (Figure 2). Despite the satisfactory compliance of the model with the experimental data, it should be emphasized that the tested material was a viscoelastic fluid. This meant that, apart from viscous features, they were also characterized by elastic properties, which may also affect the flow of chitosan sol. In order to determine the influence of the viscoelastic properties on the potential increase in flow resistance, studies to verify the possibility of describing chitosan systems using the Cox–Merz rule were carried out. According to this rule, the apparent viscosity value obtained during rotational measurements is equal to the value of complex viscosity determined from oscillation measurements for the same deformation value, shear rate, and angular frequency. Such a phenomenon, and thus the fulfillment of the Cox–Merz rule, are observed for most melted polymers and their solutions.

**Figure 7:**
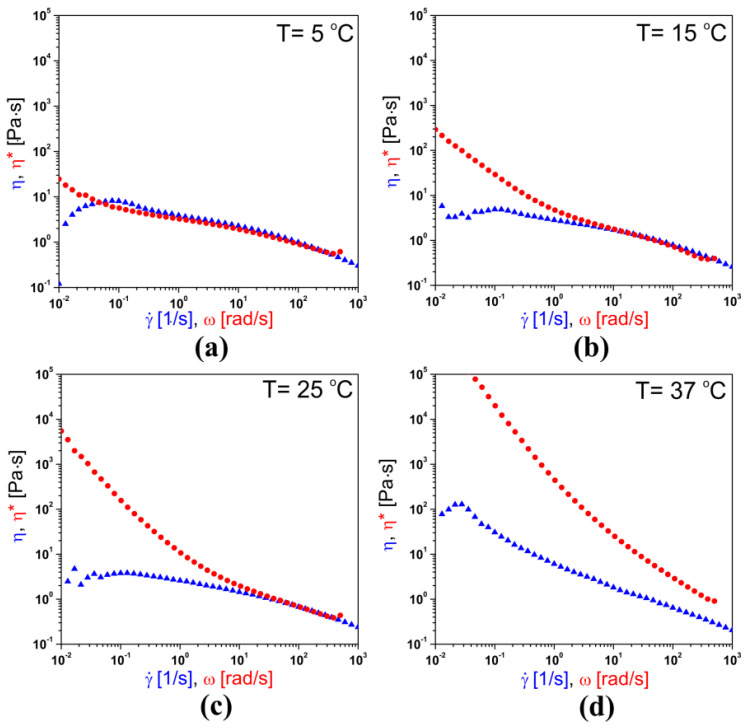
Experimental curves of dynamic viscosity and complex viscosity changes as a function of shear rate and angular frequency, respectively, at (**a**) 5 °C, (**b**) 15 °C, (**c**) 25 °C, and (**d**) 37 °C.

The results of the tests conducted at subsequent temperatures are shown in Figure 7. On their basis, it can be stated that at the lowest temperature, i.e., 5 °C, the Cox–Merz rule was fulfilled in a wide range of applied deformations, from 0.8 rad·s^−1^ to 400 rad·s^−1^. However, as the temperature increased, the range of converging the experimental curves of dynamic viscosity and complex viscosity became narrower.

From the administration point of view, the most interesting observation was made during the analysis of the course of the experimental curves obtained at 25 °C (Figure 7c). It could be seen that the dynamic viscosity and complex viscosity reached the same values for shear rates above 30 s^−1^. This meant that in the range of the shear rate values observed in injection needles, the viscoelastic properties did not increase the viscosity of the fluid. Consequently, the chitosan sol can be successfully described using only the shear-thinning behavior determined by the parameters of the Ostwald–de Waele model.

The narrowing of the Cox–Merz rule applicability range observed with the increase of temperature resulted from the progressive aggregation of colloidal chitosan systems. In line with the gelation mechanism proposed in the authors’ previous works [15,52], an increase in temperature in the initial stage of aggregation leads to a gradual growth of unstable embryos. When using low angular frequency values, they will exhibit an elastic response to the applied deformation. When the critical deformation values of approximately 5 rad·s^−1^ at 15 °C and 30 rad·s^−1^ at 25 °C are exceeded, the shear-induced deformation of the chitosan molecules will occur. Under these conditions, the flow of chitosan sol will be determined only by the nonlinear (viscous) response [53] to the applied stress; consequently, the flow of the fluid gel [54] may occur. Finally, at 37 °C, the experimental curves had a completely different course, which indicated the failure to meet the Cox–Merz rule, resulting from enhanced physical interactions leading to the formation of an infinite, three-dimensional polymer matrix. During this process, an increase in the dominance of elastic features over viscous ones associated with the formation of a highly cross-linked structure was observed. Thus, the results obtained during measurements at higher temperatures differed from the data presented for acid chitosan solutions. Martinez-Ruvalcaba et al. [55] showed that chitosan solutions without the addition of disodium β-glycerophosphate salts meet the Cox–Merz rule up to 45 °C.

**Figure 8:**
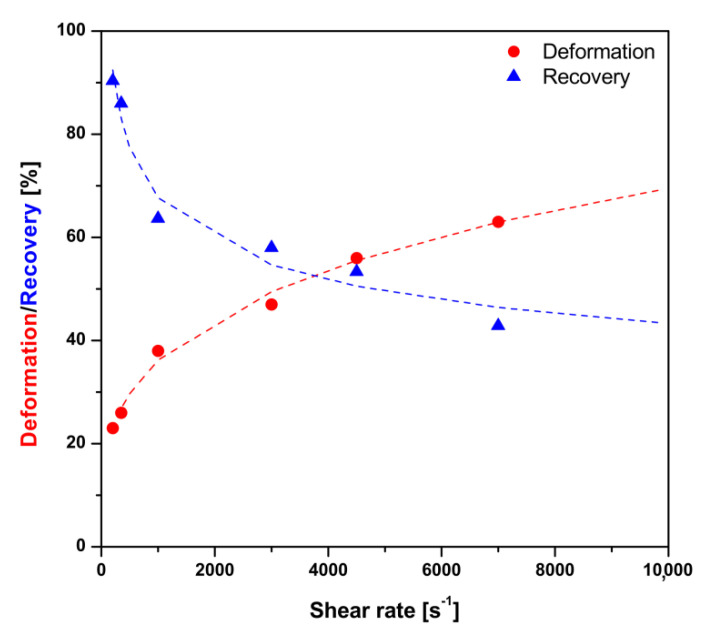
Dependence of deformation and recovery parameters as a function of the applied shear rate.

In order to determine the influence of the flow of chitosan sol through the injection needle on their macroscopic structural properties, three-interval thixotropic tests were carried out in the oscillatory-rotational-oscillatory shear intervals. The values of deformation and recovery parameters were determined based on changes in the storage modulus G’ value in accordance with Equations (1) and (2); their dependence as a function of the applied shear rate is shown in Figure 8.

The obtained values of the degree of deformation as well as the recovery of the polymer structure clearly indicated strong thixotropic properties of the studied material. It was found that the increase in the shear rate caused a strong deformation of chitosan molecules and hindered the reconstruction of the internal structure. It was found that the dependence of the obtained parameters on the applied shear rate could be described by the power function. It is worth noting that the dependence of the deformation parameter as a function of the shear rate reflected the course of the flow curve of the shear thinning fluid. However, this parameter was calculated based on the changes in the value of the storage modulus G’ which determines the elastic properties of the chitosan sol. Being careful, the determined power model can be used to determine the degree of deformation and recovery of the experimental material even outside the measuring range of the instrument, i.e., for values of shear rates above 20,000 s^−1^, which may occur when using the smallest injection needles.

## 4. Discussion

The colloidal chitosan systems obtained with the use of a long-chain unbranched polymer exhibited a shear-thinning behavior. This resulted from the well-known phenomenon in the polymer rheology of the disentanglement of polymer chains and their ordering along the shear field. This phenomenon was also observed for thermosensitive chitosan colloids and described in the previous research of the authors using rheometric measurements combined with small-angle light scattering technique [46]. This remark is very important since the considered systems must form a polymer network after injection application; changes in their conformation during the flow through the needle may affect both the phase transition conditions and the final mechanical properties of the obtained scaffolds. These assumptions were confirmed by the results of the three-interval thixotropic tests. The application of an intensive, short-term interval of rotational shear caused a strong deformation of the experimental medium, and its recovery was much more difficult. Although the tested material exhibited viscoelastic properties, in the shear rate ranges observed in injection needles at room temperature, they were characterized by equal values of apparent viscosity and complex viscosity. Thus, the Cox–Merz rule was met under the considered conditions, and the tested colloidal system was characterized by a nonlinear (viscous) response to the applied deformation, causing the flow of chitosan sol with ordered polymer chains along the shear field. This meant that when analyzing the injectability process, there was no need to consider the elastic properties of the investigated fluid. The model used to predict the forces necessary for injection based solely on the shear-thinning nature of the chitosan sol satisfactorily described the obtained experimental data.

Particular attention should be paid to the experimental data obtained in determining the effect of uncontrolled storage of chitosan sol at room temperature on their injectability. It appeared that storing the sample for up to 1 h reduced the injection force values. This resulted from the decrease in viscosity observed during spontaneous heating of the medium from the actual storage temperature (5 °C). Although this phenomenon may raise doubts, in the case of thermosensitive systems undergoing phase transition with increasing temperature, it should be emphasized that the actual process of forming an unlimited structure is preceded by a viscoelastic flow region.

Based on the experimental data obtained, it can be clearly stated that the main injection resistance is related to the flow of the medium through the injection needle; the smaller the needle (capillary) diameter used, the greater the flow resistance (linear pressure drops). In theoretical considerations of the injection process, especially when using large needles, the flow of chitosan sol through the syringe should also be considered. Simultaneously, based on the theoretical analysis of the local resistances related to the rapid narrowing of the diameter during the inflow into and outflow of the needle may be neglected due to the very small values.

## 5. Conclusions and Outlook

In the presented work, a comprehensive theoretical and experimental analysis of the injectability process of thermosensitive chitosan gels was carried out. It should be noted that the applied equation for predicting the injection force value showed very good agreement with the experimental data on the widest range of injection needles considered so far, where the flow conditions determined by the shear rate value varied in the range of 200 s^−1^ < y < 55,000 s^−1^. Simultaneously, it had been shown that in the considered flow conditions, it was not necessary to take into account the elastic properties of the chitosan sol.

Due to their strongly shear-thinning nature, the considered systems are a promising material for use as injectable scaffolds, the application of which is possible with very thin needles (even 23G and 25G), thus limiting their invasiveness and reducing patients’ discomfort.

It was found that the increase in the shear rate value led to macroscopic structural changes of the chitosan sol caused by the disentangling and ordering of the polysaccharide chains along the shear field. Therefore, it seems justified to conduct further research involving the influence of the injection application on the sol–gel phase transition conditions as well as the mechanical properties of the obtained hydrogels, which may be significantly determined by the conformation of the polymer chains during forced flow under high shear conditions.

## Figures and Tables

**Table 1:** Needles used in research.

Needle	Outer Diameter (OD) (mm)	Inner Diameter (ID) (mm)	Length (mm)	Actual Length (mm)	Actual Length/ID (-)	Shear Rate (s^−1^)	Real Shear Rate (s^−1^)	Re_MR_^1^ (−)
14G × 1½’’	2.108	1.6	40	37	23.1	186	245	0.10
15G × 1½’’	1.829	1.372	40	38	27.7	294	388	0.15
16G × 1½’’	1.651	1.194	40	41	34.3	447	589	0.22
18G × 1½’’	1.270	0.838	40	41	48.9	1292	1703	0.57
19G × 1½’’	1.067	0.686	40	41	59.8	2356	3104	0.97
20G × 1½’’	0.908	0.603	40	41	68.0	3469	4571	1.37
21G × 1½’’	0.819	0.514	40	41	79.8	5600	7380	2.09
22G × 1¼’’	0.718	0.413	30	37	89.6	10,796	14,226	3.76
23G × 1¼’’	0.641	0.337	30	37	109.8	19,871	26,185	6.49
23G × 1′’			25	31	92.0			
25G × 1′’	0.514	0.26	25	31	119.2	43,269	57,020	13.00

^1^ Value of the generalized Reynolds number proposed by Metzner and Reed, determined based on Equation (5).

**Table 2:** The set value of the crosshead speed (mm·s^−1^) in order to obtain the assumed shear speed in the needle.

Needle	Assumed Shear Rate at the Injection Needle (s^−1^)			
	700	3400	13,500	27,000
15G	1.8			
16G	1.2			
18G	0.4	2.0		
19G		1.1		
20G	0.2		3.0	
21G	0.09	0.5	1.9	
22G				1.9
23G			0.5	1
25G				0.5
